# In Vitro Analysis of TGF-β Signaling Modulation of Porcine Alveolar Macrophages in Porcine Circovirus Type 2b Infection

**DOI:** 10.3390/vetsci9030101

**Published:** 2022-02-24

**Authors:** Shunli Yang, Muhammad Umar Zafar Khan, Baohong Liu, Muhammad Humza, Shuanghui Yin, Jianping Cai

**Affiliations:** 1 State Key Laboratory of Veterinary Etiological Biology, National Foot and Mouth Disease Reference Laboratory, Lanzhou Veterinary Research Institute, Chinese Academy of Agricultural Sciences, Lanzhou 730046, China; yangshunli@caas.cn (S.Y.); umarzafar815@gmail.com (M.U.Z.K.); liubaohong@caas.cn (B.L.); 2 Department of Veterinary Sciences, Superior University, Lahore 54000, Pakistan; 3 Institute of Food Science and Technology, Chinese Academy of Agricultural Sciences (CAAS), Beijing 100193, China; muhammadhumza1992@outlook.com; 4 Jiangsu Co-Innovation Center for Prevention and Control of Important Animal Infectious Diseases and Zoonoses, Yanzhou 225009, China

**Keywords:** porcine circovirus 2, transforming growth factor-beta, mRNA, signaling pathway analysis

## Abstract

Porcine circovirus 2 (PCV2) has been recognized as an immunosuppressive pathogen. However, the crosstalk between this virus and its host cells in related signaling pathways remains poorly understood. In this study, the expression profiles of 84 genes involved in transforming growth factor-beta (TGF-β) signaling pathway were probed in PCV2b-infected primary porcine alveolar macrophages (PAMs) by using an RT^2^ profiler PCR array system. The protein expression levels of cytokines involved in the TGF-β signaling pathway were determined with a RayBiotech fluorescent Quantibody^®^ porcine cytokine array system. Results showed that 48, 30, and 42 genes were differentially expressed at 1, 24, and 48 h after infection, respectively. A large number of genes analyzed by a co-expression network and implicated in transcriptional regulation and apoptosis were differentially expressed in PCV2b-infected PAMs. Among these genes, TGF-β, interleukin-10, CCAAT/enhancer-binding protein beta (C/EBPB), growth arrest, and DNA-damage-inducible 45 beta (GADD45B), and BCL2 were upregulated. By contrast, SMAD family member 1 (smad1) and smad3 were downregulated. These results suggested that the TGF-β signaling pathway was repressed in PAMs at the early onset of PCV2 infection. The inhibited apoptosis was indicated by the upregulated C/EBPB, GADD45B, and BCL2, and by the downregulated smad1 and smad3, which possibly increased the duration of PCV2 replication-permissive conditions and caused a persistent infection. Our study may provide insights into the underlying antiviral functional changes in the immune system of PCV2-infected pigs.

## 1. Introduction

Porcine circovirus 2 (PCV2), a non-enveloped, single-stranded circular DNA virus, is the primary causative pathogen of PCV2 systemic disease (PCV2-SD) and other PCV2 associated diseases (PCVADs). PCVADs are the most economically important diseases affecting the global swine industry [1,2]. A remarkable feature of PCV2-SD pigs is systemic immunosuppression. Pigs with PCV2-SD show severe depletion in lymphocytes in their lymphoid tissues and a remarkable decrease in T and B cells in their peripheral blood, suggesting that lymphoid depletion largely contributes to immunosuppression in PCV2-affected pigs [3,4,5]. The PCV2-induced decrease in the proliferation and lysis of lymphocytes [6,7] and apoptosis of primary lymphoid organs or precursor cells [8,9,10], are potential causes of lymphocyte depletion.

Cytokines, secreted by cells undergoing cellular stress, are essential for the development of the immune response. The differential expression of some cytokines, such as interleukin (IL)-1/8/10/12, and TNF-α, in PCV2-infected porcine monocytes and macrophages, have been reported [11,12,13,14,15]. IL-10 has been suggested to play an important role in PCV2-induced systemic immunosuppression. Transforming growth factor-beta (TGF-β) and its signaling pathway participates in cell growth, apoptosis, differentiation, migration, and metastasis in a context-dependent manner in various cell types [16,17]. Dysregulation of TGF-β signaling pathway has been implicated in numerous human diseases [18]. In addition, it is a pleiotropic cytokine and has regulatory activity on multiple types of immune cells. T cells are established as critical targets of TGF-β, which regulates T cell development, homeostasis, tolerance, and differentiation [19,20,21]. TGF-β plays its biological role primarily through the canonical Smads signaling pathway which has three isoforms that are involved in several developmental processes as TGF-βs, TβRs, and Smads [22]. Current reports indicated that the TGF-β and IL-10 play an important role in immunosuppression induced by porcine reproductive and respiratory syndrome virus (PRRSV) [23,24,25,26]. However, the involvement of TGF-β and its regulated downstream events in PCV2-induced systemic immunosuppression in PCV2-infected pigs is yet to be investigated. In this study, the early expression profile of TGF-β and its signaling pathway targets in porcine alveolar macrophage (PAM) inoculated with PCV2 were analyzed using a PCR array system. Our results will enhance our understanding of the role of TGF-β and its signaling pathway targets in the interaction of immune cells and PCV2.

## 2. Materials and Methods

### 2.1. Animals and Isolation of PAM Cells

Three one-month-old Landrace pigs free of PCV1, PCV2, swine fever virus, and PRRSV, as confirmed by PCR and serum antibody testing, were housed at the Pig Unit of the Laboratory Animal Center of Lanzhou Veterinary Research Institute.

Before the collection of alveolar fluid, piglets were euthanized with intravenous sodium pentobarbital overdose. PAMs were isolated from lungs according to previously described methods [27,28]. The isolated PAMs was adjusted to 1 × 10^7^ cells/mL and cultured with a growth medium containing RPMI-1640 supplemented with 10% (*v/v*) fetal bovine serum (FBS, Gibco, Thermo Fisher Scientific Inc., Waltham, MA, USA), 100 U/mL penicillin, and 100 μg/mL streptomycin and incubated at 37 °C with 5% CO_2_.

### 2.2. Virus Preparation for Infection

PCV2b virus (ShDNY-PCV2, GenBank No. FJ948167, https://www.ncbi.nlm.nih.Gov/nuccore/FJ948167.1/, accessed on 11 August 2010) was propagated in porcine kidney 15 cell line (PK-15) as described previously [28]. PK-15 cells monolayers were inoculated at 60% confluence. After 24 h of incubation at 37 °C, the monolayer was treated with 200 mM D-glucosamine for 1 h. After further 72 h incubation, the cells were harvested and lysed three times by freeze-thawing. The genomic copies of the PCV2 in lysed cell mixture were determined by qPCR as previously described [29]. 

PAMs were infected with PCV2b in multiplicity of infection (MOI) 1 (1 viral DNA copy/cell) as described previously [28]. After infection, cells were cultured in a maintained medium RPMI-1640 supplemented with 2% FBS and incubated at 37 °C, and 5% CO_2_, for 1, 24, and 48 h respectively. The cells and supernatant were collected respectively at indicated time intervals. The cells were kept in RNAlater^®^ RNA stabilization reagent kits (Qiagen, Hilden, Germany) to prevent RNA degradation and stored at −70 °C until use.

### 2.3. Detection of PCV2 in PAMs

PCV2 in PAMs after the infection was detected through immunofluorescence assay (IFA) [30]. The cells were fixed with 4% paraformaldehyde (Solarbio^®^, Beijing, China) and permeabilized with 0.1% TritonX-100 in PBS (Sigma, Burlington, MA, USA). PCV2 in PAMs was indicated by Quantum dots conjugated specific single domain antibody (QDs-psdAb) probe.

### 2.4. RNA Extraction and cDNA Preparation

Total RNA was extracted by using an RNA extraction kit (Qiagen, Hilden, Germany) according to the instruction. The quantity of RNA was determined with a microspectrophotometer (GE, Boston, MA, USA), and the RNA integrity was evaluated through 1.8% agarose gel electrophoresis. Up to 2 μg of total RNA was reverse transcribed to cDNA using an RT^2^ HT first strand kit (Qiagen, Hilden, Germany) according to the instruction. cDNA was stored at −20 °C for further quantitative PCR (qPCR) assay.

### 2.5. RT^2^ Profiler PCR Array Assay

TGF-β signaling pathway-related genes were detected by using RT^2^ Profiler PCR Array kits (PASS-235Z, Qiagen, Hilden, Germany) in the Mx3005p real-time PCR cycler (Agilent Technologies, Santa Clara, CA, USA) as described previously [28]. The mRNA expression levels of aim genes were analyzed using the 2^−ΔΔCt^ methods by PCR Array system software version 3.5 (Qiagen, Hilden, Germany).

### 2.6. Analysis of TGF-β Expressions

The secreted TGF-β in the supernatant of PAMs post PCV2 infection was detected by Quantibody^®^ porcine cytokine array system (RayBiotech, Guangzhou, China) as described previously [28].

### 2.7. Co-Expression Network Analysis

The expression data of 84 genes were subjected to co-expression network analysis. A gene co-expression network was built based on a gene co-expression measure by the R programming language. Pearson’s correlation coefficient was used for all possible gene pairs, and the corresponding *p*-value was calculated for each correlation. A total of 717 links pairs yielded *p* < 0.01. The network was visualized in Cytoscape 3.1.1 [31,32], and network topology was examined with the Network Analyzer tool in Cytoscape. MCODE, a Cytoscape plugin [33], was utilized to identify co-expression modules for the whole network.

### 2.8. Statistic Analysis

Statistical differences for PCR array and cytokine array data were evaluated by Student’s *t*-test. A *p*-value of less than 0.05 was considered significant.

## 3. Results

### 3.1. Confirmation of PCV2b Infection in PAMs

**Figure 1:**
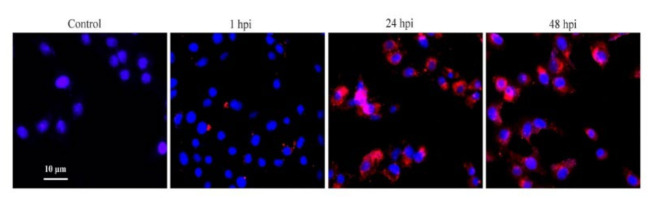
PCV2 infection in PAMs. PCV2 in PAMs post-infection was detected by IFA based on the ODs-psdAb probe (red), the nucleus was stained with DAPI (blue).

PCV2b-infected PAMs were monitored for the presence of PCV2 by IFA. IFA revealed the presence of virions of PCV2b in PAMs at 24 and 48 hpi compared with mock-infected control (Figure 1).

### 3.2. MRNA Expression Profiles

**Figure 2:**
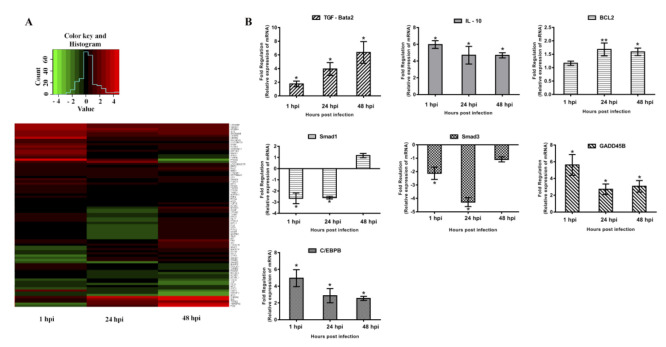
Gene expression patterns. Sixty-three target genes involved in the TGF-β signaling pathway were differentially expressed and were significantly different between PCV2-infected and control PAMs (**A**). Differentially expressed genes at each time point (**B**). Green: negative fold change level. Red: positive fold change level. Fold change was obtained by comparing the relative mRNA expression of target genes in PCV2-infected and uninfected control PAMs. Results are representative of three independent experiments and are presented as mean ± SE. ** represents *p* <0.01, * represents *p* < 0.05.

To investigate the roles of TGF-β and its regulated downstream events in the interaction of PCV2 with host cells, PCV2b-infected PAMs were analyzed by using a PCR array kit. In this study gene expression differences between the control and treatment groups were considered significant using *p* < 0.05 and an absolute fold change ≥1.5 as a cut-off [34,35]. The fold change of differential gene expression is shown with the color scheme in the heatmap shown in Figure 2A. A total of 58 target genes involved in the TGF-β signaling pathway were differentially expressed (*p* < 0.05) in different stages of infection between the control and PCV2-infected groups (Table 1). At 1 hpi, 35 genes were upregulated, and 13 genes were downregulated. At 24 hpi, 21 genes were upregulated, and 9 genes were downregulated. At 48 hpi, 29 genes were upregulated, and 13 genes were downregulated.

In these differentially expressed genes, the immunosuppressive cytokines, TGF-β and IL-10 were upregulated significantly at 24 and 48 hpi. Smad family member 1 (smad1) and smad3, key mediators of the TGF-β signaling pathway, were downregulated significantly at 1 and 24 hpi. CCAAT/enhancer-binding protein beta (C/EBPB), growth arrest and DNA-damage-inducible 45 beta (GADD45B), and BCL2, which are associated with cell proliferation and apoptosis, were upregulated at different time points after PCV2 infection (Figure 2B).

### 3.3. Expression Analyses of TGF-β in PCV2b-Infected PAMs

**Figure 3:**
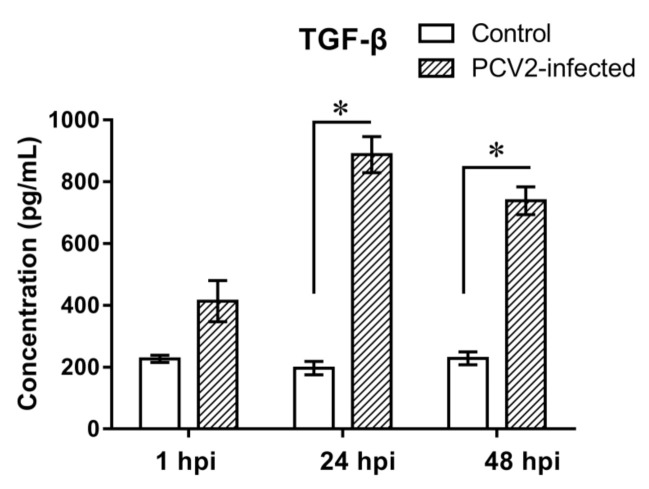
Expression of TGF-β at the protein level. TGF-β in the culture supernatants of control and PCV2-infected PAMs were measured by using porcine cytokine array kits. Data are expressed as pg/mL of three independent experiments. * represents *p* < 0.05.

PCR array results showed that TGF-β was upregulated significantly in PAMs post PCV2b infection. To further examine the expression in protein level, TGF-β in a culture supernatant of PAMs post PCV2b infection was detected by the Quantibody^®^ porcine cytokine array kits. Results showed that the TGF-β was upregulated significantly in protein levels, which was consistent with the detected by PCR array (Figure 3).

### 3.4. Network Modularity

**Figure 4:**
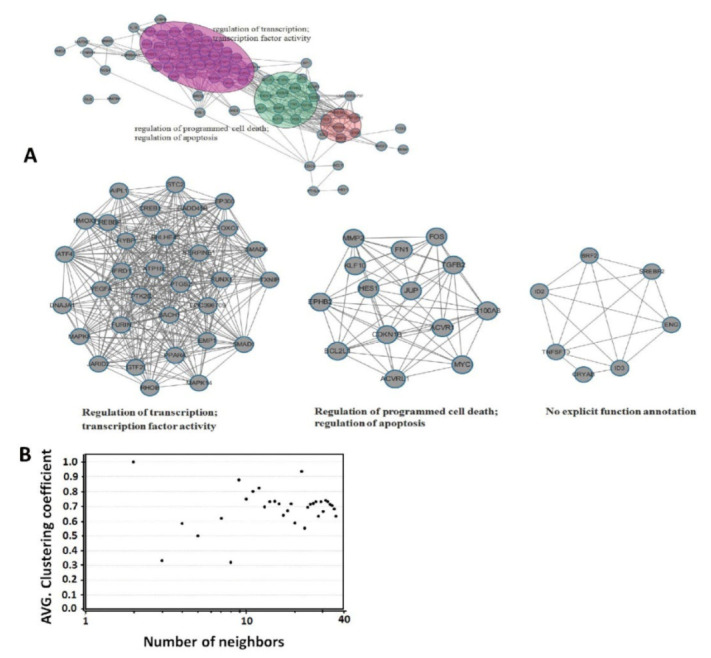
Co-expression network. A co-expression network from the expression profiles of 84 TGF-β signaling pathway-related genes was constructed. Interactive modules (**A**), Clustering coefficients (**B**).

We constructed the co-expression network from the expression profiles of 84 TGF-β pathway-related genes. A total of 717 links were found in the network between the 84 genes, which were clustered to three highly interactive modules (Figure 4A) and showed high average clustering coefficients (Figure 4B). Functional analysis showed that the largest module had 32 enriched genes for the regulation of transcription. For these genes, 24 were upregulated and 5 were downregulated at 1 hpi, 7 were upregulated and 1 was downregulated at 24 hpi, and 10 were upregulated, and 5 were downregulated at 48 hpi. The second module with 14 genes was involved in the regulation of programmed cell death. For these genes, 3 were upregulated and 5 were downregulated at 1 hpi, 5 were upregulated and 5 were downregulated at 24 hpi, and 8 were upregulated, and 5 were downregulated at 48 hpi. The third module with seven genes had no explicit functional annotation. The gene expression levels of ID2 (2.99 fold at 48 hpi), ID3 (6.84 and 28.03 fold at 24 and 48 hpi), and SREBF2 (−1.9 and −5.81 fold at 24 and 48 hpi), which are implicated in the regulation of transcription, were significantly altered. TNFSF10, the apoptosis-related gene, was downregulated at 1 hpi but was upregulated at 24 and 48 hpi (Table 1).

## 4. Discussion

PCV2, one of the most important pathogens in domestic swine worldwide, is the causative agent of PCVAD, a widespread complex multi-factorial disease with recognized immunosuppressive characteristics. Vaccines against this virus display high levels of efficacy in controlling the infection [36,37], but the molecular mechanisms of immunosuppression in PCVAD remain largely unknown. PCV2 infection and replication in immune system cells are known as the potential mechanism for virus-induced immunosuppression. In addition, PCV2 causes immunosuppression by induction of apoptosis of immune system cells, and by acting as cytokines or cytokines inhibitors/enhancers [5,38]. Previous reports have shown that TGF-β is involved in apoptosis [39,40] and cell survival [41]. The present study aimed to analyze the expression profile of TGF-β and its signaling pathway target genes in the interaction of PCV2 with PAMs.

Monocyte/macrophage lineage cells play important roles in resisting the infection of pathogens. PCV2 has been shown to infect and persist in cells of the immune system, including PAMs [42,43,44]. PCV2 alone likely causes remarkable functional impairment in PAMs, including a reduction in phagocytosis and microbicidal capability [43]. Transcription analyses on PCV2-infected monocyte/macrophage lineage cells have shown that genes related to inflammation and apoptosis are differentially expressed [14]. In the present study, TGF-β signaling pathway target genes were analyzed in PCV2-infected PAMs by using a PCR array system. Meanwhile, the functions of 84 TGF-β signaling pathway target genes were explored by a co-expression network analysis. These genes were clustered into three highly interactive modules, which showed that their functions are in the regulation of transcription, programmed cell death, and apoptosis. The results of the PCR array showed that 58 of the 84 genes targeting the TGF-β signaling pathway were differentially expressed in PAM in different stages of post PCV2 infection. These results suggested that the TGF-β signaling pathway was possibly implicated in functional changes in PCV2-infected PAMs. Interestingly, 48 genes were differentially regulated at 1 hpi which only satisfy the adsorption and internalization for the virus [45]. Our results indicated that PAMs showed a strong reaction toward PCV2 during adsorption and internalization of the virus. Moreover, results suggested that the nucleic acid and structural protein of PCV2 may play a vital role in PAM’s reactivity. However, a comparative analysis of the differentially expressed genes in different infection stages revealed reduced host response of PAMs to PCV2-challenge. In particular, the genes involved in the regulation of transcription were reduced at 1 hpi (24 upregulated and 5 downregulated) to 48 hpi (10 upregulated and 5 downregulated). PAMs are the first line of defense cells to encounter PCV2 in the lungs and are involved in phagocytic clearance, inflammatory reactions, and tissue homeostasis. Thus, a decreased response to PCV2 may be a result of self-regulation of PAMs. 

Whether gene expression leads to protein expression changes was determined by analyzing the differentially expressed genes TGF-β at the protein level with a Quantibody^®^ array assay. The difference in TGF-β expression fitted well at mRNA and protein levels. Systemic immunosuppression is a remarkable feature of PCV2-infected pigs. TGF-β, which affects proliferation, differentiation, and apoptosis of immune cells through its signaling pathway, is also an immunosuppressive cytokine. In this study, TGF-β were upregulated significantly in PAM post-PCV2 challenge, implying that they may function in PCV2 pathogenesis. 

TGF-β was significantly upregulated at mRNA and protein levels in PAM post-PCV2 infection. Smad1 and smad3, however, key mediators of the TGF-β signaling pathway for cell apoptosis [46,47,48] were downregulated. Moreover, BCL2, a hallmark protein participating in TGF-β-induced apoptosis and functioning pathophysiologically in apoptosis protection [49,50], was upregulated. In addition, C/EBPB and C/EBPB were upregulated. C/EBPB is involved in the differentiation of macrophages to functionally different subpopulations, and it is a key regulator for the survival of alveolar macrophages [51,52,53]. GADD45B, an anti-apoptotic factor, represses JNK-mediated apoptosis [54] and enhances the activation of C/EBPB. These two factors can function cooperatively in the regulation of cell differentiation [55]. Thus, the upregulation of BCL2, C/EBPB, and GADD45B, along with the downregulation of smad1 and smad3 may be a mechanism of apoptosis inhibition in PCV2-infected PAMs. These results suggested that even though TGF-β increased, PCV2 infection may have repressed TGF-β signaling pathway-induced apoptosis in PAMs. The inhibition of apoptosis will prolong the life of PAMs and may, therefore, contribute to PCV2 escaping host immunity and lengthen the duration of PCV2 replication-permissive conditions.

Global gene expression in PCV2-infected PAMs based on microarray analysis has been reported previously [14,56]. Similar to described for microarray analysis on PCV2-infected PAMs at an early stage (24 and 48 hpi), PCV2 seems to induce a large number of genes differential expression. Particularly, our research highlight the expression trend of EMP1, TNFSF10, S100A8, BCL2, and MYC genes involved in the regulation of transcription and apoptosis were the same as the microarray analysis [56]. However, the differentially expressed genes in this study, such as C/EBPB, CREBBP, IL-10, GADD45B, ID3, TGF-β (Table 1) were not shown in the differential expressed gene data of microarray analysis [14,56]. In addition, Mavrommatis et al. research [14] showed that PCV2 infection of PAMs (1 and 24 hpi) resulted in differential regulation of much fewer genes than described here or in Li et al. [56]. Whereas the differences between these results are not completely clear. The use of the different mock-infection, virus strains, and multiplicity of infection may be contributing to the differences between these results. Indeed, the virus strains and multiplicity of infection were different in these studies. Moreover, the treatment of cells and the reagents contaminated by LPS were potential reasons as well [14]. Furthermore, the different pig breeds (large white piglets, crossbred large white × landrace piglets were used in Li et al. [56] and Mavrommatis et al. [14] research respectively, landrace piglets was used here) and the potential microorganisms in PAMs (even though the specific pathogens are free) that may contribute to differential regulation of these genes.

## 5. Conclusions

PCR array analysis showed that 58 of the downstream genes of the TGF-β signaling pathway were differentially expressed in PAMs in different stages after PCV2 infection. Among these genes, IL-10 and TGF-β were upregulated significantly, suggesting their participation in PCV2 pathogenesis. However, the inhibited apoptosis in the PAMs indicated by the downregulated smad1 and smad3 and by the upregulated C/EBPB, GADD45B, and BCL2 may have partly prolonged PCV2 replication-permissive conditions and caused persistent infection in clinical cases. The observed expression profile of TGF-β signaling pathway-associated genes in PCV2-infected PAMs may provide a possible mechanism of the functional changes in the immune system of PCV2-affected pigs.

## Figures and Tables

**Table 1:** Differentially expressed TGF-β target genes (*p* < 0.05 and absolute fold change ≥1.5) between PCV2-infected and mock-infected control. Numbers in bold denote significant differential expression.

Gene Symbol	Description	Fold Change		
		1 hpi	24 hpi	48 hpi
Regulation of Transcription and Transcription Factor Activity				
C/EBPB	CCAAT/enhancer binding protein (C/EBP), beta	4.96	2.87	2.55
CREBBP	CREB binding protein	8.75	4.33	3.66
HMOX1	Heme oxygenase (decycling) 1	7.53	4.49	3.50
IL10	Interleukin 10	5.97	4.69	4.68
GADD45B	Growth arrest and DNA-damage-inducible, beta	5.62	2.71	3.07
NFKBIA	Nuclear factor of kappa light polypeptide gene enhancer in B-cells inhibitor, alpha	8.67	5.95	3.99
RYBP	RING1 and YY1 binding protein	3.42	1.59	1.54
ID3	Inhibitor of DNA binding 3, dominant negative helix-loop-helix protein	1.28	6.84	28.03
RUNX1	Runt-related transcription factor 1	5.78	1.03	1.76
MBD1	Methyl-CpG binding domain protein 1	1.95	1.49	1.65
SERPINE1	Serpin peptidase inhibitor, clade E (nexin, plasminogen activator inhibitor type 1), member 1	4.17	–1.12	1.93
ID2	Inhibitor of DNA binding 2, dominant negative helix-loop-helix protein	1.84	–1.01	2.99
ATF4	Activating transcription factor 4 (tax-responsive enhancer element B67)	2.08	1.26	1.15
ATP1B1	ATPase, Na+/K+ transporting, beta 1 polypeptide	5.83	1.49	−1.22
BACH1	BTB and CNC homology 1, basic leucine zipper transcription factor 1	5.48	1.06	−1.04
BHLHE40	Basic helix-loop-helix family, member e40	2.76	1.32	1.43
FOXO1	Forkhead box O1	1.85	1.15	1.33
PTK2B	PTK2B protein tyrosine kinase 2 beta	1.79	−1.01	−1.01
TXNIP	Thioredoxin interacting protein	1.55	−1.28	1.19
VEGFA	Vascular endothelial growth factor-A	12.49	1.52	1.91
MAPK8	Mitogen-activated protein kinase 8	1.54	−1.01	−1.31
IFRD1	Interferon-related developmental regulator 1	3.87	1.04	−1.25
LOC396709	Retinoic acid receptor alpha	2.89	1.15	1.17
HSP90AA1	90-kDa heat shock protein	1.50	1.36	1.15
LOC100620797	Focal adhesion kinase 1-like	1.39	1.39	1.79
CTNNB1	Catenin (cadherin-associated protein), beta 1, 88 kDa	1.43	1.67	1.02
SP1	Sp1 transcription factor	−1.01	1.52	1.41
PTGS2	Prostaglandin G/H synthase-2	21.61	2.25	−5.99
RGS4	Regulator of G-protein signaling 4	2.39	7.21	−2.49
FURIN	Furin-like	2.50	−1.18	−1.57
JARID2	Jumonji, AT rich interactive domain 2	1.63	−1.12	−1.74
EMP1	Epithelial membrane protein 1	3.35	−1.52	−3.65
TNFSF10	Tumor necrosis factor (ligand) superfamily, member 10	−4.98	3.95	13.72
MAPK14	Mitogen-activated protein kinase 14	−1.95	1.67	1.63
STC2	Stanniocalcin 2	−2.06	−1.57	−1.52
ME2	Malic enzyme 2, NAD(+)-dependent, mitochondrial	−1.63	−1.18	−1.60
GTF2I	General transcription factor IIi	−3.86	−1.07	1.34
PPARA	Peroxisome proliferator-activated receptor alpha	−3.69	1.41	1.39
Regulation of programmed cell death and apoptosis				
TGFB2	Transforming growth factor, beta 2	1.76	3.94	6.36
S100A8	S100 calcium binding protein A8	2.91	14.55	20.75
SMAD6	SMAD family member 6	2.10	−1.01	1.44
BCL2L1	BCL2-like 1	1.16	1.68	1.59
CDKN1B	Cyclin-dependent kinase inhibitor 1B (p27, Kip1)	−1.22	1.73	2.20
FOS	FBJ murine osteosarcoma viral oncogene homolog	−2.63	2.37	18.83
FN1	Fibronectin 1	1.74	−1.12	3.78
MMP2	Matrix metallopeptidase 2 (gelatinase A, 72kDa gelatinase, 72kDa type IV collagenase)	−1.96	1.23	2.88
ACVR1	Activin A receptor, type I	−1.15	−1.58	−1.82
ACVRL1	Activin A receptor type II-like 1	−1.18	−1.69	−1.78
MYC	V-myc myelocytomatosis viral oncogene homolog (avian)	−1.39	−3.56	−6.66
HES1	Hairy and enhancer of split 1, (Drosophila)	−1.33	−2.15	−3.09
JUP	Junction plakoglobin	−1.30	1.00	−3.28
SMAD1	SMAD family member 1	−2.66	−2.60	1.17
SMAD3	SMAD family member 3	−2.12	−4.28	−1.08
EPHB2	EPH receptor B2	−1.00	−1.73	−1.21
No explicit function annotation				
CRYAB	Crystallin, alpha B	−1.25	−2.25	1.56
SREBF2	Sterol regulatory element binding transcription factor 2	−1.41	−1.90	−5.81
ENG	Endoglin	1.58	1.39	2.08
BRF2	Zinc finger protein 36, C3H type-like 2	1.33	1.21	1.93

## Data Availability

Materials described in the manuscript are freely available to any scientist wishing to use them.
